# The value of serum tumour markers in the prediction of aetiology and follow up of patients with pericardial effusion

**DOI:** 10.5830/CVJA-2011-029

**Published:** 2012-04

**Authors:** U Bildirici, U Celikyurt, E Acar, T Sahin, G Kozdag, D Ural, O Bulut

**Affiliations:** Department of Cardiology, Kocaeli University Medical Faculty, Kocaeli, Turkey; Department of Cardiology, Kocaeli University Medical Faculty, Kocaeli, Turkey; Department of Cardiology, Kocaeli University Medical Faculty, Kocaeli, Turkey; Department of Cardiology, Kocaeli University Medical Faculty, Kocaeli, Turkey; Department of Cardiology, Kocaeli University Medical Faculty, Kocaeli, Turkey; Department of Cardiology, Kocaeli University Medical Faculty, Kocaeli, Turkey; Seka State Hospital, Kocaeli, Turkey

**Keywords:** pericardial effusion, CA 125, CA 15-3, CEA

## Abstract

**Background:**

The aim of this study was to evaluate the value of tumour markers in the differential diagnosis of pericardial effusions and to assess their changing levels during follow up.

**Methods:**

Sixty-nine patients who were admitted to hospital with a diagnosis of pericardial effusion were included in the study. Serum tumour markers were measured on admission and after a mean of 18 ± 7 months’ follow up. An aetiological diagnosis was made on clinical evaluation, imaging techniques and biochemical, microbiological and pathological analysis. The patients were divided into five groups according to the aetiology of their pericardial effusions.

**Results:**

Carbohydrate antigen (CA) 12-5 and CA 15-3, and carcinoembryonic antigen (CEA) levels were significantly higher in patients with malignancies than in those with viral/idiopathic pericarditis. With multivariate analysis, CA 15-3 levels were found to be the most significant determinant (*p* = 0.027). In the ROC curve analysis, CA 15-3 values above 25 U/ml predicted a malignancy with 71% sensitivity and 78% specificity.

**Conclusion:**

Tumour markers, particularly CA 15-3, may be useful in the differential diagnosis and prediction of malignancies in patients with pericardial effusion. In patients with viral/idiopathic aetiology, these serum tumour markers were slightly elevated in the acute phase, but after a mean of one year of follow up, their levels returned to normal, contrary to those with malignancies.

## Abstract

Pericardial effusions (PE) are common and produced by a wide variety of diseases,^01^,^02^ including viral and bacterial infections, tuberculosis, malignancy, heart failure, chronic renal failure and rheumatic diseases.^01^,^02^ However, despite all the diagnostic tests available, the most common cause is idiopathic.^01^-^03^ Malignancy is not the main cause of PE, but PE may be the first indication of cancer, and therefore early detection would enable rapid diagnosis, which is important in improving the survival rate of cancer patients.^04^

Sampling of pericardial fluid (pericardiocentesis) and pericardial biopsy play an important role in identifying the underlying aetiology of PE.^05^ It is not always possible to perform pericardial biopsy, however, due to its potential complications. Although pericardiocentesis is easier to perform than pericardial biopsy, it has a low probability of detection of malignant cells (30–50%).^06^ High complication rates in mild and moderate effusions also limit its diagnostic application.

There are a limited number of markers available for the evaluation and differential diagnosis of PE,^06^,^07^ including carbohydrate antigen (CA) 125, CA 15.3 and carcinoembryonic antigen (CEA).^06^,^08^ CEA has been identified as a useful marker for differentiating PE related to malignant pathology of the gastrointestinal system.^05^,^09^,^10^

Increased levels of CA 15-3 in the blood are primarily observed in breast cancer.^11^,^12^ CA 15-3 has also be used to evaluate both pleural and pericardial effusions.^13^-^16^ CA 125 levels may be detected in pericardial fluid secretions related to both malignant and benign aetiologies.^16^,^17^ Therefore, the prognostic value of CA 125 for the detection of malignancies is limited.^18^

The aim of this study was to evaluate the relationship between tumour markers and the underlying aetiology in patients with PE. We also examined the diagnostic value of these tumour markers in detecting malignancies in patients with PE, and determined their changing levels during follow up.

## Methods

A total of 76 patients with PE who were admitted or referred to our hospital between January 2004 and March 2007 were included in the study. The mean follow-up period was 18 ± 7 months (range 8–27 months). The aetiological evaluation included complete blood count, measurement of troponin I, erythrocyte sedimentation rate, viral disease determination (Epstein Barr, cytomegalo virus, coxsackie A virus, parva virus, hepatitis A, B, C), thyroid-stimulating hormone, and rheumatological markers [rheumatoid factor (RF), anti-nuclear antibody, anti-smooth muscle antibody, anti-double-stranded DNA].

All patients underwent computerised tomography of the thorax. Patients with heart failure (ejection fraction < 45%) and severe pericardial effusion were excluded (as it could have affected the marker levels). Patients who refused follow-up visits were also excluded. In total, seven patients were excluded from the study.

Echocardiography was performed on all patients and CA markers were checked at the time of hospitalisation and at the end of the follow-up period. Pericardiocentesis was not performed because the patients had only mild or moderate pericardial effusions and there was no sign of compression of the heart.

CEA, CA 15-3 and CA 19-9 levels were measured in blood samples using an electrochemiluminescence immunoassay on a Roche Modular E170. CA 125 levels were measured with a quantitative immunoassay technique. All results were evaluated by a biochemist who did not know the patients.

Echocardiographic examinations were performed by an experienced cardiologist who was blinded to the tests. They were performed using standard protocol and a standard device (Vivid 7, GE Medical Systems, Horten, Norway). Measurement of the cardiac walls was done according to the American Society of Echocardiography guidelines. Since the number of patients with PE was not large enough to establish quantitative amounts of fluid, these were arbitrarily established by echocardiography using the sum of the epicardial and pericardial separations in the anterior and posterior spaces.^19^ The effusions were graded as mild (less than 10 mm), moderate (10–20 mm) and severe (more than 20 mm).^19^

## Statistical analysis

SPSS Inc for Windows, standard version 11.0 was used for statistical analysis. All data are given as mean and standard deviation. Comparison between patients with malignant and other aetiologies was done using the Student’s *t*-test, and for unequally distributed variables, the Mann-Whitney *U*-test. Logistic regression analysis adjusting for CA 125, CA 15-3, CA 19-9, alpha-fetoprotein (AFP), prostate membrane antigen (PSA) and CEA was done to evaluate the relationship between malignancies and tumour markers. Threshold values of tumour markers were established by ROC analysis.

## Results

A total of 69 patients (32 women and 37 men with an average age of 58 ± 17 years) were included in the study. Aetiological diagnosis was done on clinical evaluation, imaging techniques and biochemical markers. The patients were grouped into categories depending on the aetiology of the PE (group 1: viral/idiopathic; group 2: bacterial; group 3: tuberculosis; group 4: malignancy; group 5: other).

Twenty-one patients were found to have malignancies and 48 had benign aetiologies after the first examination. During the follow-up period, cancer (three lymphoma, one thymoma, one lung cancer, one gastrointestinal malignancy) developed in six patients who had been considered idiopathic at the first examination. Finally, we had 42 patients with benign and 27 with malignant aetiologies (11 lymphoma, six breast cancer, five lung cancer, three thymoma, one ovarian cancer, one gastrointestinal malignancy). The aetiology in 27 patients could not be identified and they were considered as the viral/idiopathic PE group.

**Fig. 1:**
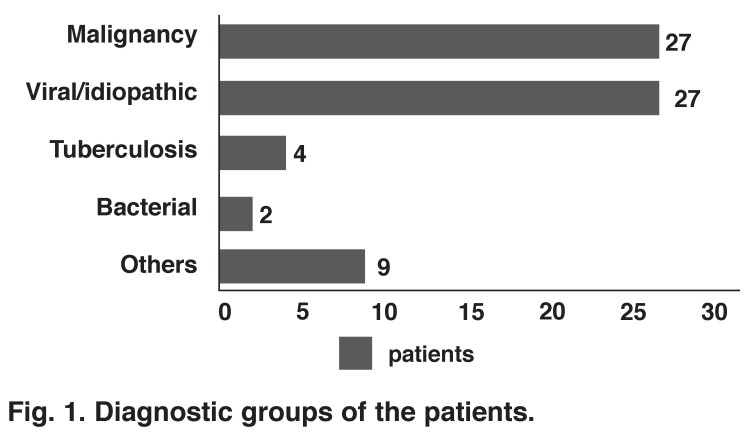
Diagnostic groups of the patients.

Aortic dissection (one patient), tuberculosis (four patients), bacterial microorganism (two patients), autoimmune or rheumatic disease (three patients), chronic renal disease (four patients) and coronary bypass graft operation (one patient) were benign underlying causes of PE in 15 patients (Fig. 1). The characteristics of the patients with malignancies and those with other aetiologies of PE are shown in Table 1. The clinical and echocardiographic characteristics of the patients are shown in Table 2. Fibrin bands were detected most commonly in patients with tuberculosis (2: 50%), however, this was statistically insignificant.

**Table 1:** Characteristics Of Patients With PE Of Malignant And Benign Aetiologies

	*Malignant PE*	*Benign PE*	p
Patient (M/F)	27 (14/13)	42 (23/19)	NS
Age (years)	57.7 ± 17.8	52.1 ± 13.9	NS
Glucose (mg/dl)	123 ± 50.2	121.7 ± 46.3	NS
Urea (mg/dl)	48 ± 22.9	39.2 ± 22.9	NS
Creatinine (mg/dl)	1.1 ± 0.4	1.3 ± 0.7	NS
Sedimentation (mm/h)	83.2 ± 40.4	67.6 ± 37.9	NS
WBC × 1 000	18.4 ± 19.3	20.6 ± 21.9	NS
CRP (mg/dl)	14.5 ± 11.2	10.5 ± 9.1	NS
Duration of hospitalisation (days)	28.3 ± 11.4	17.3 ± 9.7	NS
Treatment type			
NSAID	14	20	
Corticosteroid	3	3	
NSAI + colchicine	10	19	

NSAID; non-steroid anti-inflammatory drug, WBC; white blood cell, M; male, F; female, NS; not significant.

**Table 2:** Echocardiographic Parameters Of Patients With PE Of Malignant And Benign Aetiologies

	*Malignant PE (n = 27)*	*Non-malignant PE (n = 42)*	p
LVEDD	48.1 ± 6.1	47.4 ± 5.3	NS
LVESD	12.1 ± 1.7	11.6 ± 2.1	NS
PWD	11.3 ± 2.3	11.1 ± 1.5	NS
EF	64.5 ± 15.6	63.1 ± 17.2	NS
LAD	39.6 ± 5.9	36.2 ± 7.3	NS
E	1.2 ± 0.9	1.2 ± 0.7	NS
RA	1 ± 0.8	1.1 ± 0.6	NS
RV	24.7 ± 2.9	24.1 ± 4.1	NS

LVEDD; left ventricular end-diastolic dimension, LVESD; left ventricular end-systolic dimension, PWD; posterior wall dimension, EF; ejection fraction, LAD; left atrial diameter, E; mitral early flow, RA; right atrium, RV; right ventricle; NS; not significant.

Levels of CA 125, CA 15-3 and CEA in the group with malignancies were significantly higher than in the group with no malignancies (Table 3). No significant differences were detected between the levels of CA 19.9, PSA and AFP. The relationship between malignant aetiology and tumour markers was evaluated with multivariate analysis, adjusting for CA 125, CA 15-3 and CEA. The only significant relationship was observed with CA 15-3 (0.027). ROC analyses were applied to detect the threshold levels of tumour markers for detecting effusions of malignant origin.

**Table 3:** Tumour Marker Levels Of The Patients With And Without Cancer

	*Malignant PE (n = 27)*	*Non-malignant PE (n = 42)*	p
CA 125 (U/ml)	90.3 ± 88.6	55.2 ± 67.8	0.03
CA 15-3 (U/ml)	32.3 ± 14.6	17.0 ± 8.9	0.002
CA 19-9 (U/ml)	18.9 ± 20.2	18.7 ± 19.4	NS
CEA (ng/ml)	4.5 ± 5.2	2.0 ± 1.2	0.04
AFP (U/ml)	1.9 ± 1.7	1.7 ± 0.8	NS
PSA (mg/ml)	2.1 ± 2.5	3.1 ± 4.1	NS

NS; not significant.

In the ROC curve analyses, a CA 15-3 level above 25 U/ml had a sensitivity of 71% and specificity of 78% for predicting pericardial effusions caused by malignancies (AUC = 0.83, SE = 0.05, *p* = 0.002). Elevated levels of the three markers (cut-off for CA 125 = 66 U/ml; CA 15-3 = 25 U/ml; CEA = 4.2 U/ml) had a sensitivity of 69% and specificity of 88% for the prediction of pericardial effusions caused by malignancies.

In the follow-up period, levels of CA 125 and CA 15-3 decreased significantly in the patients in the idiopathic/viral group. Levels of CA 125 also decreased significantly in the patients in the malignancy and tuberculosis groups. However CA 15-3 levels remained constant in the group of patients with malignancies. Levels of CEA did not change significantly in any group. Levels of CA 125 were significantly higher at the beginning, but this significance decreased in the follow-up period.

During the follow-up period, malignancy was detected in six patients in the idiopathic group (three lymphoma, one thymoma, one lung cancer, one gastrointestinal malignancy). CA 125 and CA 15-3 levels were high in five and three patients, respectively.

## Discussion

In this study, we examined the diagnostic value of the CA 19-9, CA 125, CEA, CA 15-3, AFP and PSA for the diagnosis of tumour aetiology in patients with PE. The levels of CA 15-3, CEA and CA 125 were significantly higher in PE patients with malignancies. In the follow-up period, the levels of CA 15-3 and CA 125 decreased in patients in the idiopathic/viral group and remained constant in those with malignancies. The levels of CA 15-3 were more significant in detecting malignancies than those of CA 125.

CEA levels are known to increase in heart failure and this marker has also been used to diagnose pleural effusions with malignant aetiologies.^10^ In many studies, a relationship has been found between high levels of CEA and pericardial effusions with malignant aetiologies.^05^,^20^,^21^ Szturmowicz *et al.* found CEA levels above 5 U/ml had a 90% specificity for the detection of malignancy.^05^ Similarly, in our study, the levels of CEA were significantly higher in patients with cancer. In the follow-up period, this significance did not change.

Lindgren *et al.* showed a relationship between CA 125 levels and ovarian cancer.^22^ More recently it was realised that CA 125 levels can also increase in benign serous effusions.^08^,^16^,^17^ Unlike CEA and CA 15-3, CA 125 is secreted from mesothelial cells in patients with PE of benign aetiology.^07^ For this reason, it can be used to determine the existence of fluid but it does not inform on the aetiology.^10^,^16^,^17^

Two other studies revealed that CA 125^23^ and CEA^24^ levels increased in heart failure, and that these levels could be related to the amount of pericardial fluid present.^25^ In our study, the levels of these markers were significantly higher in the PE patients with malignancies and remained high during the follow-up period.

High levels of CA 15-3 were first detected in breast cancer patients and this was used to evaluate recurrence in the follow-up period.^11^,^12^ In later studies, high levels of CA 15-3 were also found in effusion patients with malignancies.^13^ Romero *et al.* reported that CA 15-3 markers could be used to detect the underlying aetiology of the effusion.^14^

In our study, in patients with no malignancies, CA 15-3 and CA 125 levels decreased significantly in the follow-up period. The levels of CA 15-3 were significantly higher in patients with malignancies and this increase remained significant in the follow-up period. CA 15-3 was found to be a valuable marker in the multi-analysis. In ROC curve analysis, CA 15-3 levels above 25 U/ml had a 71% sensitivity and 55% specificity for the prediction of malignancies underlying the pericardial effusions.

Malignancy was developed during the follow-up period in six of our patients whose initial diagnosis was idiopathic. At least one marker was high in five of these patients and CA 15-3 levels were high in three patients. These results indicate that if the underlying aetiology of the PE is unknown but there are high levels of CEA, CA 125 and CA 15-3, one should investigate for malignancy. Use of all three markers had a low sensitivity (29%) but a high specificity (97%) for detecting malignancy. Therefore determination of levels of these markers is very useful in the early diagnosis of malignancies.

Pericardiocentesis and pericardial biopsy are the best diagnostic tests for detecting malignant aetiologies of PE. However, where pericardiocentesis is not indicated, there are a limited number of other tests available. This study indicates that CA 125, CA 15-3, CEA are useful markers for detecting malignant aetiologies of PE. High levels of all three markers or increased levels of only CA 15-3 appeared to be predictive of effusions caused by malignancy. High levels of CA 15-3 in the follow-up period supported our hypothesis. Effusions categorised as idiopathic with high levels of these markers must be followed up closely or re-examined for possible malignancies.

There were some limitations to our study. The patient numbers in the groups according to the aetiology of PE were not equal. The number of patients was limited and they were heterogenous in aetiology.

## Conclusion

CA 125, CA 15-3 and CEA markers can be used in the differential diagnosis of benign and malignant aetiologies in patients with chronic pericardial effusions. Combined use of these three markers improves their prognostic value. Importantly, we must suspect malignancy in patients with PE whose aetiology cannot be determined.
